# Dengue Virus NS1 Protein as a Diagnostic Marker: Commercially Available ELISA and Comparison to qRT-PCR and Serological Diagnostic Assays Currently Used by the State of Florida

**DOI:** 10.1155/2017/8072491

**Published:** 2017-06-27

**Authors:** Jason H. Ambrose, Shamala Devi Sekaran, Azliyati Azizan

**Affiliations:** ^1^Global Health Department, College of Public Health, University of South Florida, 12901 Bruce B Downs Blvd., Tampa, FL 33612, USA; ^2^Bureau of Public Health Laboratories, Florida Department of Health, 3602 Spectrum Blvd., Tampa, FL 33612, USA; ^3^Department of Medical Microbiology, Faculty of Medicine, University of Malaya, 50603 Kuala Lumpur, Malaysia; ^4^Nazarbayev University School of Medicine (NUSOM), 53 Kabanbay Batyr Ave., Astana 010000, Kazakhstan

## Abstract

**Background:**

The proper management of patients infected with dengue virus requires early detection. Here, real-time molecular assays have proven useful but have limitations, whereas ELISAs that detect antibodies are still favored but results are obtained too late to be of clinical value. The production of DENV NS1 peaks early during infection and its detection can combine the advantages of both diagnostic approaches.

**Methods:**

This study compared assays currently used for detecting DENV infection at the Florida Department of Health including anti-DENV IgM and IgG ELISAs as well as qRT-PCR, against a commercially available DENV NS1 ELISA. These comparisons were made among a group of 21 human sera.

**Results:**

Nine of 14 (64.3%) DENV qRT-PCR+ samples were also DENV NS1+. Interestingly, the 5 NS1− samples that were qRT-PCR+ were additionally IgM− and IgG+ suggesting a nonprimary infection. Compared to qRT-PCR, the NS1 assay had a sensitivity of 64.3%, specificity 100%, PPV of 100%, and NPV of 58.3%.

**Conclusions:**

The NS1 ELISA performed as expected in known DENV qRT-PCR+ samples; however negative NS1 results for qRT-PCR+ and IgG+ sera seemingly reduced the usefulness of the NS1 ELISA for nonprimary cases. We therefore conclude that diagnosis obtained via DENV NS1 ELISA deserves further investigation.

## 1. Introduction

Infections caused by dengue virus continue to constitute a worldwide threat to the public, both in human and in economic costs. In 2017, dengue virus remains the cause of one of the most globally significant arthropod-borne (arbo-) viral illnesses. According to WHO, there is currently an at risk global population of 3.9 billion where an estimated 390 million infections occur annually. Around 96 million infected persons seek clinical attention but the majority of cases go unreported. Approximately 500,000 of clinical patients will progress to severe illness and require hospitalization with fatalities arising in 2.5% [1, 2]. Additionally, after a 75-year absence, local transmission of DENV was documented in Florida, USA. During the time period of 2009–2012, 103 autochthonous cases were documented with the majority of those cases (27 in 2009 and 63 in 2010) associated with an outbreak of DENV serotype 1 (DENV1) in Key West (Monroe County). However, epidemiologically unrelated, locally acquired cases were also documented in Broward, Hillsborough, Miami-Dade, Palm Beach, Osceola, Martin, and Seminole counties through 2012. A second outbreak of DENV1 occurred in Martin County during 2013 [3–5]. Two introductions of dengue are thought to have occurred in Martin County, the first near Port Salerno in 2011 and the second near Jensen Beach, the second being responsible for the outbreak. In 2016, a case of DENV4* (Cone M, personal comm.)* was locally acquired in Key West [6] and dengue appeared again locally in Miami-Dade during Zika virus outbreak investigations [7].

Dengue is caused by one of four different serotypes of small RNA viruses in the family Flaviviridae, DENV1–4. The 5′ and 3′ ends of the DENV genome contain untranslated regions (UTRs) and the open reading frame first encodes the three structural proteins, C, prM/M, and env, followed by 7 nonstructural (NS) proteins, including the NS1 protein. The genome is translated as a single polyprotein that is processed and modified posttranslationally [8]. The NS1 protein itself is secreted from infected cells and is found in serum at detectable levels that overlap with peak viremia (and RNA detection). These NS1 levels also coincide with the onset of detectable IgM in acute primary cases and IgG in acute nonprimary cases [9]. It has been found that elevated levels of serum NS1 directly indicate increased viral burden and further establish the positive correlation between viremia and NS1 profiles [10, 11]. NS1 is a generally conserved protein among flaviviruses but has been found to contain both cross-reactive and serotype-specific epitopes among dengue viruses; these are important factors when considering development of immunoassays [12–14]. For these reasons NS1 is considered as having diagnostic value as a viral marker of infection. The protein is found both intracellularly and in a soluble form (sNS1) secreted from infected host cells but its function remains enigmatic. The immature form of NS1 is that of a monomer that is variably glycosylated but readily forms heat-labile homodimers usually associated with the surface of infected cells [8, 14]. From there, the major oligomeric form of sNS1 is thought to be a hexamer of around 300 kDa. The hexamer consists of 3 dimeric subunits that are noncovalently bound and are less stable than NS1 dimers [15, 16].

Dengue is a problematic disease to manage at the clinical level, in large part due to late manifestations of severe illness in some patients [17]. In the past, techniques including virus isolation and serological assays such as ELISA and plaque-reduction neutralization assays (PRNT) typically yielded results after clinical resolution (or development of severe illness), leading to diagnosis with no benefit to the patient. However, the capabilities of the laboratory have advanced to the level of obtaining same day results in acutely infected patients with the advent of rapid techniques that include molecular diagnostic assays such as real-time qRT-PCR [9]. As alluded to above, the NS1 protein of DENV is also a useful early viral marker of infection and an ELISA (Panbio® Dengue Early ELISA #E-DEN02P) that detects it is currently available from Inverness Medical (now Alere Inc.) among other manufacturers of similar immunoassays. These DENV NS1 immunoassays may represent the new paradigm for DENV diagnosis in many parts of the world, in part, by serving to combine the pros of both traditional serological assays and those of modern molecular assays. These include early diagnosis commensurate with the capabilities of molecular assays coupled with decreased costs in both equipment and reagents along with the reduction of the technical prowess and stringency required for performing them in the clinic. This study attempted to ascertain the potential value of the DENV NS1 ELISA detailed Section 2.3 in diagnosing dengue in the US state of Florida.

## 2. Materials and Methods

### 2.1. Ethics Statement

The removal of identifiers in these previously collected serum samples resulted in the determination of this study as not meeting the definition of human research activities and thus IRB exempt under US 45 CFR 46.101(4). This ruling was determined by the University of South Florida IRB.

### 2.2. Sample Selection

A series of 21 serum samples previously assayed at the Florida Department of Health- (FLDOH-) Bureau of Public Health Laboratories- (BOPHL-) Tampa for DENV by qRT-PCR and either anti-DENV IgM and IgG ELISA in concert (20/21) or IgM only (1/21) were subsequently subjected to DENV NS1 detection by ELISA. Corresponding DENV serotypes of positive samples were also obtained via qRT-PCR. Eight of 14 qRT-PCR+ samples were positive for DENV1, five were DENV4+, and one was DENV2+. No samples that were identified as DENV3+ were included in this study. Dengue qRT-PCR− samples belonged to either of the following diagnostic categories: anti-DENV IgG+ (*n* = 2) or samples negative for all routine DENV diagnostic assays. The latter samples were obtained from either the clinical archive (*n* = 2) or a serosurvey (*n* = 3) conducted in Martin County, Florida, during the course of an outbreak of DENV1. The DENV IgG and IgM ELISAs were adapted from protocols provided by CDC-Arboviral Diseases Branch (Ft. Collins, CO) and TaqMan-based DENV serotype-specific qRT-PCR was performed using an FDA-approved protocol provided by CDC-Dengue Branch (San Juan, Puerto Rico).

### 2.3. DENV NS1 ELISA

The Panbio dengue early ELISA (Inverness Medical, Sinnamon Park, QLD, Australia, #E-DEN02P) was used to determine the presence of DENV NS1 in individual serum samples. Each sample was run in duplicate, according to manufacturer's instructions with the following changes specific to our study. (Samples, positive controls, and negative controls were added to wells in duplicate and calibrators were added in quadruplicate, all at 100 *μ*L. The ELISA sample plates were read at 450 nm with a reference filter of 620 nm. Each sample OD value (absorbance) was averaged between duplicate wells and then divided by the cut-off value to obtain index values.) Index values < 0.9 were ruled as negative, between 0.9 and 1.1 as equivocal, and values above 1.1 as positive for DENV NS1 detection. The results of this assay were then compared to those of DENV virus RNA detection via qRT-PCR as well as anti-DENV IgM and IgG ELISAs previously performed at the FLDOH-BOPHL-Tampa.

## 3. Results

In our study, the DENV NS1 ELISA (Tables 1 and 2) (see Graphical Abstract in Supplementary Material available online at https://doi.org/10.1155/2017/8072491) found 9 of 14 sera to be positive for DENV that were also qRT-PCR+ and 0 of 2 that were previously positive by IgG only (9/16 total DENV+ samples). Five samples that were DENV− negative in all 3 comparison assays, including 2 clinical samples and 3 from the Martin County serosurvey, were also negative via DENV NS1 ELISA. Each of the DENV qRT-PCR+ samples that were found to be NS1 ELISA− was also negative for IgM (5/14). Interestingly, however, these 5 qRT-PCR+ samples were positive for IgG (and IgM−) by ELISA, suggesting nonprimary infection. Additionally, each of the 3 dengue serotypes represented in this study was found within this subgroup (3 of 5 samples were DENV1, 1 = DENV2, and 1 = DENV4). On the other hand, one sample that was NS1+ presented with the same profile (i.e., qRT-PCR+, IgM−, and IgG+). All seven qRT-PCR− samples were also negative for DENV NS1. Please take note that the desired direct comparison between assays was made between those capable of early detection (NS1 ELISA versus qRT-PCR). Therefore, while both assays failed to detect dengue in 2 samples that were IgG+ only, the “true negative” sample number of (*n* = 7) was left to stand for calculations so as not to skew the NPV artificially in favor of qRT-PCR. In all and when compared to the results of qRT-PCR, the NS1 assay was found to have a sensitivity of 64.3%, specificity of 100%, a positive predictive value (PPV) of 100%, and a negative predictive value (NPV) of 58.3% in this small and varied sample set (Graphical Abstract and Table 3).

## 4. Discussion

Like the results found here, previous reports of the investigational use of commercially available DENV NS1 immunoassays such as those in Brazil [18] and Malaysia [19] showed favorable results when compared against standard diagnostic methods. The former group used the Platelia™ Dengue NS1 Ag microplate EIA (Bio-Rad, Hercules, CA) and the latter the SD Bioline Dengue Duo (Standard Diagnostics, Yongin-si, Republic of Korea). These groups obtained results with sensitivities of 95.9% and 65.41% and specificities of 81.1% and 98.75%, respectively (whereby for the SD Bioline assay, a multiplex assay, only NS1 was considered). In 2010, the Platelia™ assay was approved for the screening of 80,000 Puerto Rican blood donors. At the time of FDA approval for the particular study, the test was already in use in approximately 40 countries around the world [20]. The USNIH notes on https://www.clinicaltrials.gov that the study has been completed but official results are yet to be reported [21]. In Lima et al. [22], two of the previously mentioned assays, the PanBio ELISA and the Platelia™ EIA, were compared against another immunoassay, the Dengue NS1 STRIP (Bio-Rad, Hercules, CA). The STRIP is an immunochromatographic test similar to the previously mentioned SD Bioline Dengue Duo. While all obtained specificities are near 100%, here they found the STRIP assay to have the highest sensitivity (89.6%), followed by the Platelia™ EIA at 83.6% and the Panbio ELISA at 72.3%. This group also reported that the assays were less sensitive in detecting DENV3 cases and that the Platelia™ assay detected primary cases at a statistically significant higher percentage than nonprimary cases.

It should also be noted that concerns about the sensitivity of the Platelia™ EIA arose in a study in Aracaju, Brazil, where 58 of 119 NS1 negative samples were instead found later to be DENV4+ by confirmatory tests, and their reasoning pointed to an issue with the detection of nonprimary cases [23]. As mentioned above, this was also seemingly evident in our study where all 5 NS1 ELISA− samples known to be qRT-PCR+ were also IgG+ in ELISA. However, a subsequent report published after obtaining the results reported here indicated that this drawback can be alleviated by preheating samples at 100°C for 5 m [24]. This would indicate that the assay may require dissociation of antigen-antibody complexes and/or preferentially detects monomeric NS1 over its dimeric form. The former seems very likely as, in nonprimary infections, NS1 bound by IgG antibodies produced during the early phases would reduce the pool of free and detectable serum levels of this protein. On the other hand, their data suggested that, with heating, the assay is preferentially detecting NS1 monomers in both types of dengue infection [14]. It would be important to empirically determine that the heating step reported above is producing dissociation of antigen-antibody complexes and/or dissociation of free dimeric NS1 into constituent monomers and that this step is essential for increasing sensitivity in both types of infection.

There also remains the concern that no single assay included in this study was alone sufficient for diagnosis. This was evident where 2 out of 7 qRT-PCR− samples were found to be DENV+ only via IgG detection. This in turn affected the comparison between the test under question (DENV NS1 ELISA) and the gold-standard used here for early detection (qRT-PCR). Wang and Sekaran [19] abrogated this assay-related issue to a large extent through the use of a “one-stop” rapid test able to detect not only NS1 but also IgM and/or IgG. This in turn increased the sensitivity of their combined assay and identified, concurrently, more positive individuals. This and other multifaceted approaches to DENV diagnostics seem to be the proper direction moving forward and we encourage further investigation.

Regarding our study, we accept that larger scale studies typically include greater numbers of negative samples when characterizing new assays. Here, though, both the limited number of reagents and DENV+ samples available for study compelled us to approach the study from the opposite direction. Additionally, as part of a larger study, these samples were also subject to analyses, such as immunological profiling [25] and experimental DENV NS1 detection* (Jason H. Ambrose et al., unpublished data)* further supporting the approach used here. Despite the small sample size included, we nevertheless conclude that assays detecting DENV NS1 should eventually be incorporated within the algorithms of laboratories performing dengue diagnostics, including BOPHL-Tampa. We also propose that they are investigated for further utility, especially in conjunction with not only other potential diagnostic markers, but also those of prognostic value, in order to better inform the clinic on identifying and properly managing patients infected with dengue.

## Supplementary Material

In samples obtained in Florida, the PanBio® Dengue NS1 Early ELISA detected 9/14 positive samples in those also positive for dengue in qRT-PCR. The 5 negative NS1 samples in the qRT-PCR positive subset were also positive for IgG indicating non-primary infection. No qRT-PCR negative samples were found to be NS1 positive (7/7).

## Figures and Tables

**Table 1:** *DENV NS1 detection in selected serum samples as determined by ELISA and in comparison to clinical molecular (qRT-PCR) and serological (anti-DENV IgM and IgG) results*. The table below details the results of DENV NS1 detection by ELISA against qRT-PCR, IgM, and IgG DENV assays for a group of serum samples selected for inclusion and based on the following criteria: *(1) denotes samples that were positive by qRT-PCR for DENV as determined by BOPHL-Tampa; (2) denotes samples that were positive for DENV by IgG detection only as determined by BOPHL-Tampa; (3) denotes samples that were DENV negative received by BOPHL-Tampa for all DENV-specific assays; (4) denotes samples that were collected from Martin County serosurvey and were found to be DENV negative by all DENV-specific assays*. Index values represent the mean of duplicate values obtained when reading samples at 450 nm and taking calibrators into account. Negative samples had an index value > 0.9, those between 0.9 and 1.1 were equivocal, and those above 1.1 were positive for NS1 detection. Results are listed as either positive (+) or negative (neg) for each ELISA. Positive qRT-PCR results are reported either as neg or positive by listing serotype and *C*_*T*_ value results. Samples that were qRT-PCR+ but NS1 neg are highlighted in bold font within the table. Note that no single assay here was capable of diagnosing DENV infection alone.

Sample	Index value	NS1 ELISA	qRT-PCR (*C*_*T*_)	IgM ELISA	IgG ELISA
(1-1)	5.98	+	DENV4 (20.40)	+	+
(1-2)	5.91	+	DENV1 (14.19)	Neg	Neg
(1-3)	0.08	**Neg**	**DENV1 (33.17)**	**Neg**	+
(1-5)	0.09	**Neg**	**DENV1 (34.96)**	**Neg**	+
(1-6)	5.93	+	DENV1 (26.06)	+	Neg
(1-7)	5.93	+	DENV1 (30.40)	+	Neg
(1-8)	4.51	+	DENV1 (22.32)	+	Neg
(1-9)	5.38	+	DENV1 (31.90)	+	Neg
(1-10)	0.17	**Neg**	**DENV1 (25.53)**	**Neg**	+
(1-11)	5.97	+	DENV4 (29.79)	+	+
(1-12)	5.92	+	DENV4 (20.74)	Neg	+
(1-13)	0.21	**Neg**	**DENV4 (19.69)**	**Neg**	+
(1-14)	0.25	**Neg**	**DENV2 (25.13)**	**Neg**	+
(1-15)	6.00	+	DENV4 (21.03)	+	+
(2-3)	0.43	Neg	Neg	Neg	+
(2-4)	0.13	Neg	Neg	Neg	+
(3-1)	0.05	Neg	Neg	Neg	Neg
(3-2)	0.09	Neg	Neg	Neg	Neg
(4-1)	0.05	Neg	Neg	Neg	Neg
(4-2)	0.06	Neg	Neg	Neg	Neg
(4-3)	0.06	Neg	Neg	Neg	*N/A*

**Table 2:** *Breakdown of DENV serological diagnostic status (any combination of DENV NS1, anti-DENV IgM, and/or –IgG) versus detection of DENV RNA via qRT-PCR*. The table below details first the comparison of DENV NS1 detection via ELISA compared to results obtained for the respective sample set via qRT-PCR (*n* = 21), set as a gold-standard. The table details a further breakdown of these results by including anti-DENV IgM and IgG status of the samples. Nine (9 out of 14) qRT-PCR+ samples were also DENV NS1+ (64.3%) and all 7 samples that were negative by qRT-PCR were also found to be negative for DENV NS1. Notably, all 5 DENV NS1− samples that were qRT-PCR+ were also anti-DENV IgM− and IgG+, while only 1 positive NS1 sample was found to have that same profile, indicating that nonprimary infections may affect the sensitivity of the DENV NS1 ELISA. ^*∗*^*Please note that 1 of the DENV qRT-PCR*−* samples was not assayed for DENV anti-IgG*.

DENV ELISA results	DENV qRT-PCR results versus ELISA	
	DENV qRT-PCR+ (*n* = 14)	DENV qRT-PCR− (*n* = 7)
DENV NS1+	9/14 **(64.3%)**	0/7 **(0%)**
DENV NS1−	5/14 **(35.7%)**	7/7 **(100%)**

DENV NS1+, IgM+	7/14 (50%)	0/7 (0%)
DENV NS1+, IgM−	2/14 (14.3%)	0/7 (0%)
DENV NS1−, IgM+	0/14 (0%)	0/7 (0%)
DENV NS1−, IgM−	**5/14 (35.7%)**	**7/7 (100%)**

DENV NS1+, IgG+	4/14 (28.6%)	0/6^*∗*^ (0%)
DENV NS1+, IgG−	5/14 (35.7%)	0/6^*∗*^ (0%)
DENV NS1−, IgG+	**5/14 (35.7%)**	2/6^*∗*^ (33.3%)
DENV NS1−, IgG−	0/14 (0%)	4/6^*∗*^ (66.7%)

DENV NS1+, IgM+, IgG+	3/14 (21.4%)	0/6^*∗*^ (0%)
DENV NS1+, IgM−, IgG+	1/14 (7%)	0/6^*∗*^ (0%)
DENV NS1−, IgM+, IgG+	0/14 (0%)	0/6^*∗*^ (0%)
DENV NS1−, IgM−, IgG+	5/14 (35.7%)	2/6^*∗*^ (33.3%)

DENV NS1+, IgM+, IgG−	5/14 (35.7%)	0/6^*∗*^ (0%)
DENV NS1+, IgM−, IgG−	0/14 (0%)	0/6^*∗*^ (0%)
DENV NS1−, IgM+, IgG−	0/14 (0%)	0/6^*∗*^ (0%)
DENV NS1−, IgM−, IgG−	0/14 (0%)	4/6^*∗*^ (66.7%)

**Table 3:** *Sensitivity, specificity, and positive (PPV) and negative (NPV) predictive values for Panbio DENV NS1 ELISA when compared to detection of DENV RNA via qRT-PCR*. Note that the “true negative” value of *n* = 7 was left to stand in order to prevent skewing calculations in favor of qRT-PCR even though both assays failed to diagnose dengue correctly in 2 IgG+ samples.

	DENV qRT-PCR+ (*n* = 14)	DENV qRT-PCR− (*n* = 7)	
DENV NS1 ELISA+	9	0	*PPV = 100%*
DENV NS1 ELISA−	5	7	*NPV = 58.3%*
	*Sensitivity = 64.3%*	*Specificity = 100%*	
