# Corrigendum to “Dengue Virus NS1 Protein as a Diagnostic Marker: Commercially Available ELISA and Comparison to qRT-PCR and Serological Diagnostic Assays Currently Used by the State of Florida”

**DOI:** 10.1155/2017/2046864

**Published:** 2017-10-25

**Authors:** Jason H. Ambrose, Shamala Devi Sekaran, Azliyati Azizan

**Affiliations:** ^1^Global Health Department, College of Public Health, University of South Florida, 12901 Bruce B Downs Blvd., Tampa, FL 33612, USA; ^2^Bureau of Public Health Laboratories, Florida Department of Health, 3602 Spectrum Blvd., Tampa, FL 33612, USA; ^3^Department of Medical Microbiology, Faculty of Medicine, University of Malaya, 50603 Kuala Lumpur, Malaysia; ^4^Nazarbayev University School of Medicine (NUSOM), 53 Kabanbay Batyr Ave., Astana 010000, Kazakhstan

In the article titled “Dengue Virus NS1 Protein as a Diagnostic Marker: Commercially Available ELISA and Comparison to qRT-PCR and Serological Diagnostic Assays Currently Used by the State of Florida” [1], there was an error in the legend of Table 1, where the wording “Negative samples had an index value >0.9” should be replaced by “Negative samples had an index value of less than 0.9”. The correct table and legend are as follows.

## Figures and Tables

**Table 1:** DENV NS1 detection in selected serum samples as determined by ELISA and in comparison to clinical molecular (qRT-PCR) and serological (anti-DENV IgM and IgG) results. The table details the results of DENV NS1 detection by ELISA against qRT-PCR, IgM, and IgG DENV assays for a group of serum samples selected for inclusion and based on the following criteria: (1) denotes samples that were positive by qRT-PCR for DENV as determined by BOPHL-Tampa; (2) denotes samples that were positive for DENV by IgG detection only as determined by BOPHL-Tampa; (3) denotes samples that were DENV negative received by BOPHL-Tampa for all DENV-specific assays; (4) denotes samples that were collected from Martin County serosurvey and were found to be DENV negative by all DENV-specific assays. Index values represent the mean of duplicate values obtained when reading samples at 450 nm and taking calibrators into account. Negative samples had an index value of less than 0.9, those between 0.9 and 1.1 were equivocal, and those above 1.1 were positive for NS1 detection. Results are listed as either positive (+) or negative (neg) for each ELISA. Positive qRT-PCR results are reported either as neg or positive by listing serotype and *C*_*T*_ value results. Samples that were qRT-PCR+ but NS1 neg are highlighted in bold font within the table. Note that no single assay here was capable of diagnosing DENV infection alone.

Sample	Index value	NS1 ELISA	qRT-PCR (*C*_*T*_)	IgM ELISA	IgG ELISA
(1-1)	5.98	+	DENV4 (20.40)	+	+
(1-2)	5.91	+	DENV1 (14.19)	Neg	Neg
(1-3)	0.08	**Neg**	**DENV1 (33.17)**	**Neg**	+
(1-5)	0.09	**Neg**	**DENV1 (34.96)**	**Neg**	+
(1-6)	5.93	+	DENV1 (26.06)	+	Neg
(1-7)	5.93	+	DENV1 (30.40)	+	Neg
(1-8)	4.51	+	DENV1 (22.32)	+	Neg
(1-9)	5.38	+	DENV1 (31.90)	+	Neg
(1-10)	0.17	**Neg**	**DENV1 (25.53)**	**Neg**	+
(1-11)	5.97	+	DENV4 (29.79)	+	+
(1-12)	5.92	+	DENV4 (20.74)	Neg	+
(1-13)	0.21	**Neg**	**DENV4 (19.69)**	**Neg**	+
(1-14)	0.25	**Neg**	**DENV2 (25.13)**	**Neg**	+
(1-15)	6.00	+	DENV4 (21.03)	+	+
(2-3)	0.43	Neg	Neg	Neg	+
(2-4)	0.13	Neg	Neg	Neg	+
(3-1)	0.05	Neg	Neg	Neg	Neg
(3-2)	0.09	Neg	Neg	Neg	Neg
(4-1)	0.05	Neg	Neg	Neg	Neg
(4-2)	0.06	Neg	Neg	Neg	Neg
(4-3)	0.06	Neg	Neg	Neg	*N/A*
