# Supplementary figures and images for: Dengue Virus NS1 Protein as a Diagnostic Marker: Commercially Available ELISA and Comparison to qRT-PCR and Serological Diagnostic Assays Currently Used by the State of Florida
Source: J Trop Med. 2017 Jun 27;2017:8072491. doi: 10.1155/2017/8072491 (PMC5504952; doi:10.1155/2017/8072491)

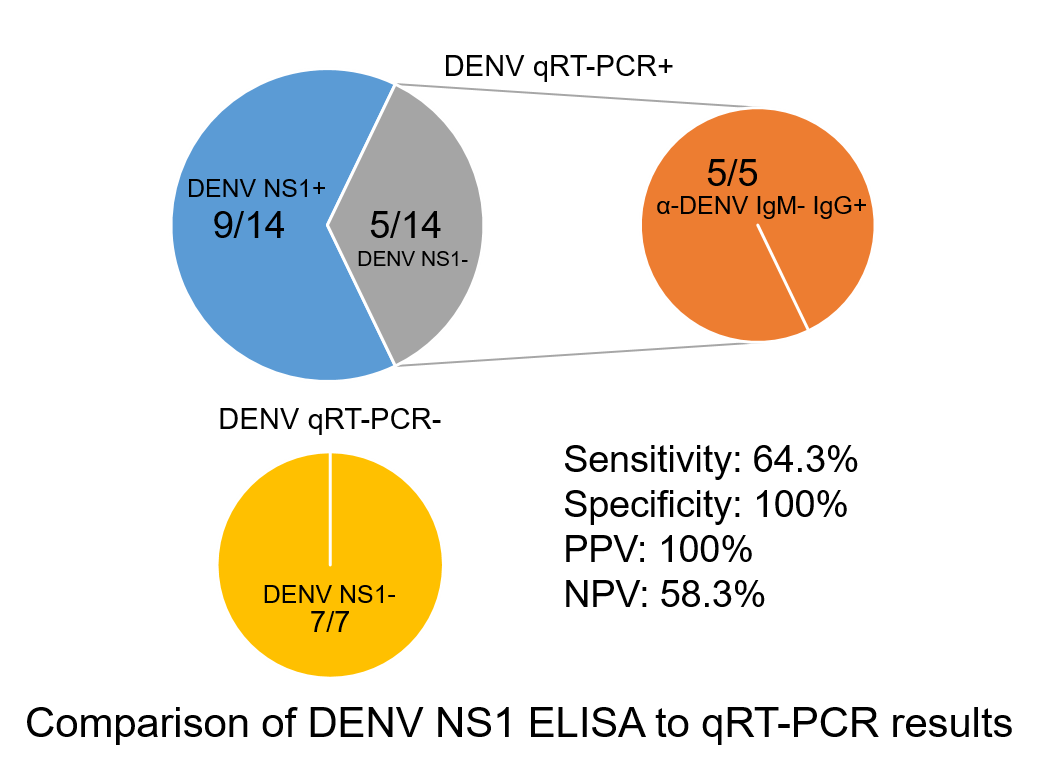

Supplement: Supplementary file 1 — In samples obtained in Florida, the PanBio® Dengue NS1 Early ELISA detected 9/14 positive samples in those also positive for dengue in qRT-PCR. The 5 negative NS1 samples in the qRT-PCR positive subset were also positive for IgG indicating non-primary infection. No qRT-PCR negative samples were found to be NS1 positive (7/7). [file 8072491.f1.tif]
